# Intravascular lithotripsy-assisted percutaneous deep vein arterialization for no-option chronic limb-threatening patients and heavily calcified tibial occlusive disease

**DOI:** 10.1177/17085381241257736

**Published:** 2024-05-28

**Authors:** Dimitrios Kapetanios, Michael Czihal, Nikolaos Tsilimparis, Giovanni Torsello, Julian Rieck, Konstantinos Stavroulakis

**Affiliations:** 1 Department of Vascular Surgery, 27192 Ludwig-Maximillians-University Hospital Munich, Munich, Germany; 2 Division of Vascular Medicine, Medical Clinic and Policlinic IV, 206621 Hospital of the Ludwig-Maximillians-University, Munich, Germany; 3 Department of Vascular and endovascular Surgery, 39612 St Franziskus Hospital Muenster, Munster, Germany; 4 Clinical Consultant and therapy development Limflow medical, Paris, France

**Keywords:** Chronic limb-threatening ischemia, intravascular lithotripsy, IVL, pDVA, percutaneous deep vein arterialization

## Abstract

**Purpose:**

To report the first chronic limb-threatening ischemia (CLTI) patients who underwent an intravascular lithotripsy (IVL)-assisted percutaneous deep vein arterialization (pDVA).

**Case Report 1:**

An 81-year-old patient presented with CLTI and a heavily calcified lesion of the popliteal artery and tibioperoneal trunk (TPT), with a distal tibial and foot arch occlusion. The patient underwent IVL and drug-coated balloon angioplasty for the distal popliteal artery and of the TPT to improve the inflow prior to pDVA. The wound situation remained stable without secondary procedure until the patient`s deaths due to complications of urosepsis 3 months later.

**Case Report 2:**

A 64-year-old patient with rest pain of the left limb with a single-vessel tibial run-off (peroneal artery) and occluded pedal arch was treated with 3.5 mm IVL followed by a successful pDVA as mentioned above. IVL performed in the proximal posterior tibial artery to optimize the inflow to the circuit and change the compliance of the crossing point from the arterial to the vein system. The patient underwent repeat angioplasty of the plantar vein arch 5 months after the index procedure and thereafter remained asymptomatic during 2 years of follow-up.

**Conclusion:**

The combined use of IVL and pDVA could improve the patency of the reconstruction with clinical benefits in no-option CTLI patients.

## Introduction

Chronic limb-threatening ischemia (CLTI) represents the most advanced stage of peripheral arterial disease.^1–3^ Conventional revascularization strategies consist of surgical and endovascular reconstruction. Nonetheless, up to 20% of CLTI patients are “non-reconstructable “due to absence of a viable distal target vessel for a bypass grafting or endovascular treatment.^4^ Thus, percutaneous deep venous arterialization (pDVA) might serve as an alternative in selected high risk for amputation patients.

Besides the distal tibial occlusions, severe calcification might further complicate the revascularization of CLTI patients. It is estimated that 30%–50% of patients with peripheral atherosclerosis have a significant vascular calcification, which is a negative predictor for the outcome of surgical and endovascular treatment.^5,6^ In cases of pDVA, the presence of heavily calcified disease might challenge the crossing from the arterial to the vein system, leading to inadequate stent graft expansion and occlusions. Intravascular lithotripsy (IVL) has been both used for the modification and treatment of calcified disease. Accordingly, the combination of pDVA and IVL might be useful in patients with heavily calcified disease and distal tibial and foot arterial occlusion. We report the first two cases of IVL-assisted pDVA with the LimFlow system.

### Case Report 1

**Image 1.**
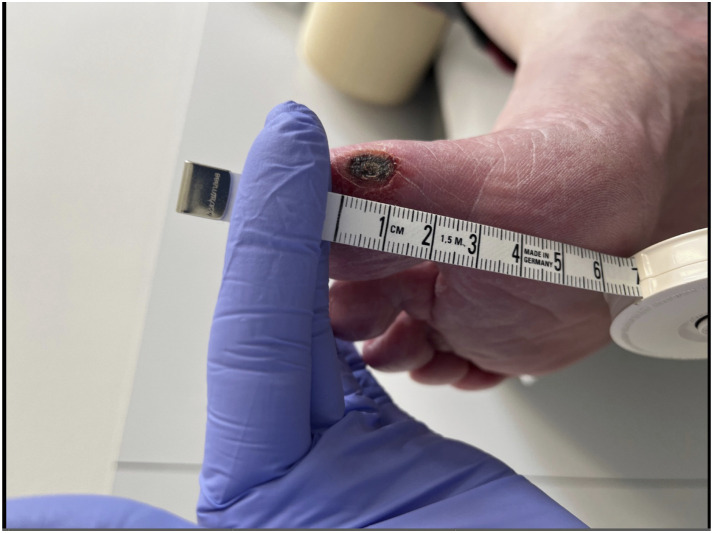
Preprocedural image showing the foot lesion of case report 1.

An 81-year-old male patient was submitted with rest pain and gangrene of the right first toe (Image 1). The patient had significant comorbidities including diabetes mellitus, coronary heart disease, non-dialysis-dependent chronic kidney disease, and arterial hypertension, and he was a previous smoker (42 pack years). A below-the-knee amputation of the contralateral limb was already performed.

A diagnostic angiography showed severely calcified stenosis of the distal popliteal artery and the tibioperoneal trunk (TPT) and distal tibial artery occlusion with only collateral flow to the foot. An endovascular recanalization of the anterior tibial artery was attempted but was unsuccessful. Due to absence of a distal target artery for surgery, the patient was assessed for pDVA.

**Figure 1.**
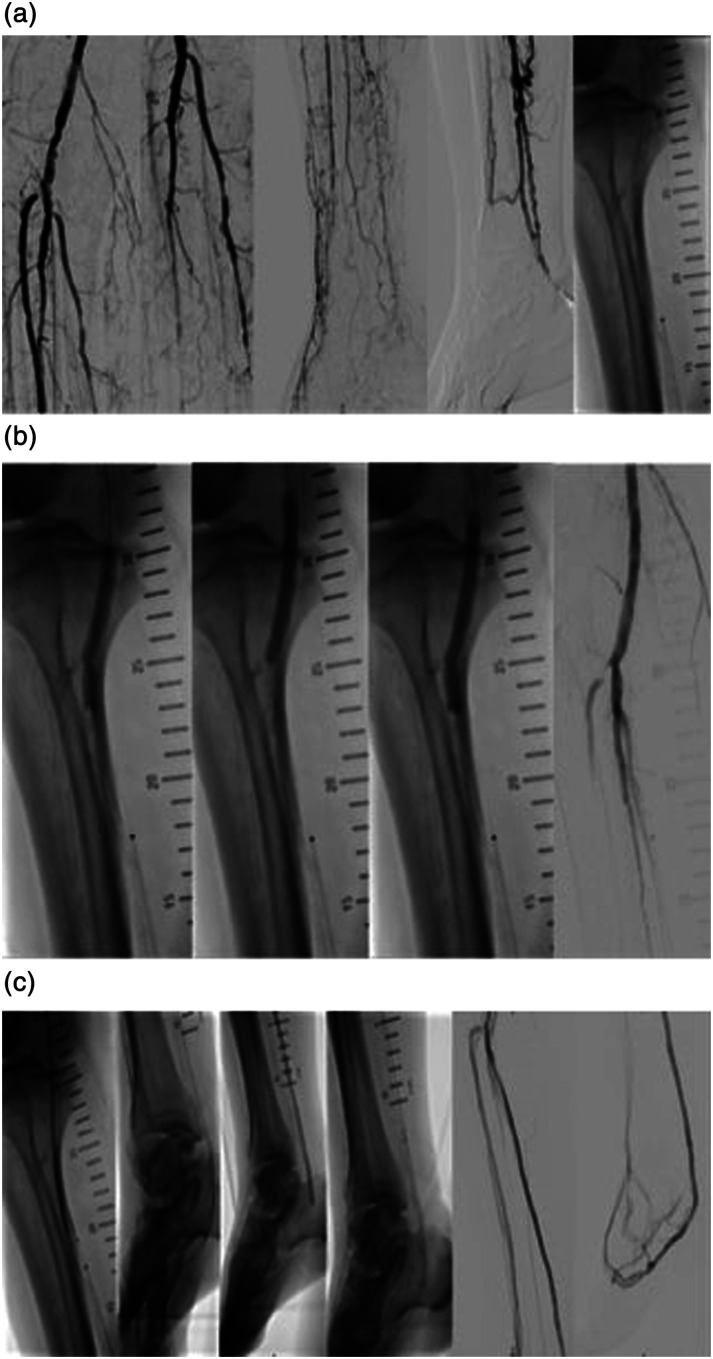
Intravascular lithotripsy-assisted percutaneous deep vein arterialization. (a) Diagnostic angiogram and arterial and vein access. (b) Intravascular lithotripsy and drug-coated balloon angioplasty for the distal popliteal artery and the tibioperoneal trunk. (c) Percutaneous deep vein arterialization.

A detailed description of the LimFlow pDVA has been described elsewhere.^7^ The procedure was conducted in a hybrid operating room under general anesthesia. First, retrograde venous access was obtained, and a 4F 45 cm sheath was advanced up to the proximal posterior tibial vein. Afterward, a 7 F antegrade arterial sheath was introduced. Following the diagnostic imaging, a 0.014″ glidewire advantage guidewire (Terumo inc.) and a 0.035″ Navicross catheter (Terumo inc.) were used to cross the calcified lesions of the distal popliteal artery and the TPT. Both vessels were predilated with an undersized plain old balloon angioplasty (POBA) catheter (3 mm Coyote, Boston Sci) to allow the delivery of the IVL catheter. A 6.5 mm IVL device (M5+, Shockwave Medical) was then used to treat the severely calcified stenosis of the distal popliteal artery and the TPT. A drug-coated balloon (DCB) angioplasty (6 mm Ranger, Boston Sci) was then applied as anti-restenotic treatment. The pDVA was completed based on the instructions for use for the LimFlow system. Following crossing and valvulotomy, the posterior vein was dilated with a 5 mm balloon catheter and the LimFlow straight covered stents were deployed (three 5.5 × 100 mm stents and one 5.5 × 60 mm stent) from the level of the calcaneus to the crossing point. A tapered covered stent (3.3 to 5 mm × 60 mm) was then used to bridge the crossing point. DCB angioplasty with a Stellarex 5 × 60 mm Balloon was conducted at the distal end of the deployed grafts to avoid an edge stenosis. The final angiography showed a good result with rush blood flow through the stents and through the pedal veins with outflow through the vein arch (Figure 1). The postoperative course was uneventful. During 4 months of follow-up, the patient did not undergo any secondary procedure or minor/major amputation and rest pain relieved as ongoing patency of the pDVA was controlled with regular clinical and color duplex ultrasound examinations. Four months after the procedure, the patient died secondary to complications of urosepsis.

### Case Report 2

A 64-year-old male patient presented with rest pain of left foot. The patient suffered from long-standing diabetes mellitus and from non-dialysis-dependent chronic kidney disease. A diagnostic angiogram showed a single-vessel run-off through the peroneal artery and no patent foot arch vessels (desert foot). The proximal posterior and anterior tibial arteries exhibited stenotic and heavily calcified arteriosclerosis, followed by long segment occlusions. After failed antegrade/retrograde revascularization attempt of the anterior and posterior tibial arteries, a pDVA was indicated.

**Figure 2.**
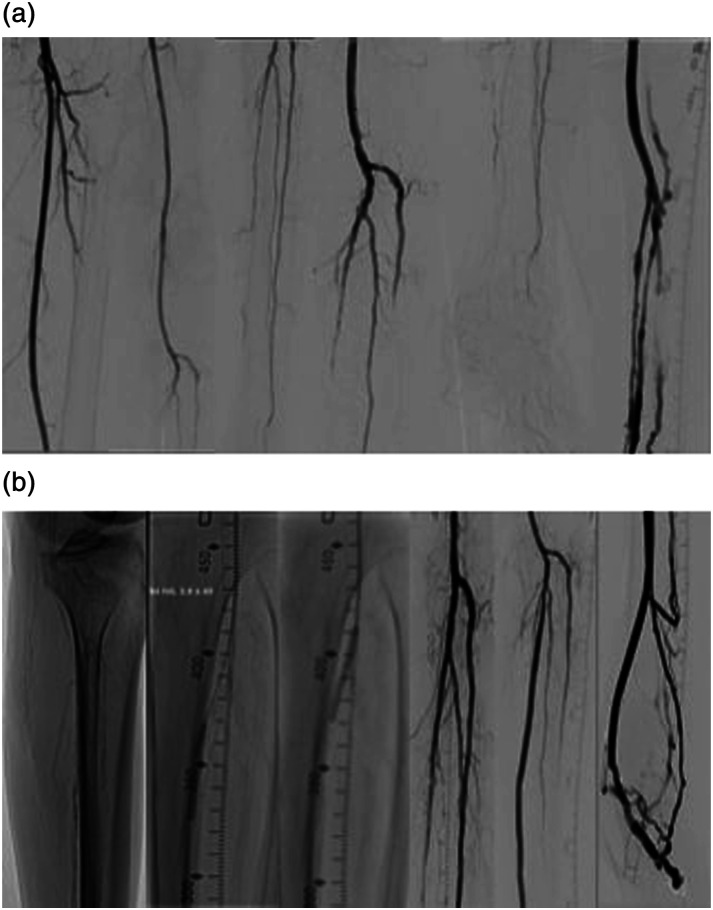
Intravascular lithotripsy (IVL) to optimize the inflow and change the compliance of the crossing point prior to percutaneous deep vein arterialization (pDVA). (a) Diagnostic angiogram and retrograde access and (b) IVL of the proximal tibial vessels and the crossing point and pDVA.

**Figure 3.**
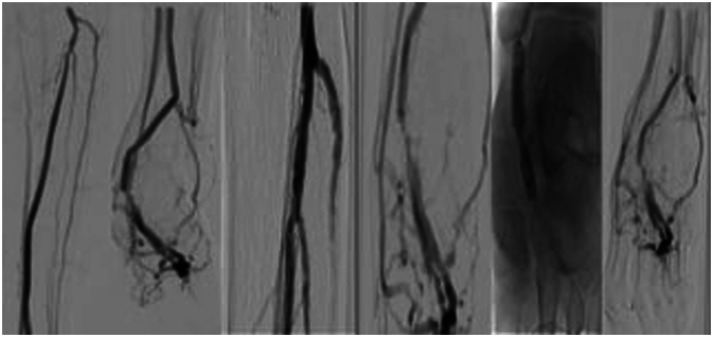
Target lesion revascularization of the vein foot arch, without stenosis of the proximal reconstruction, 3 months after the index procedure.

Similar to the aforementioned procedure, the pDVA was performed in the hybrid operation room under general anesthesia. After obtaining a retrograde vein access and an antegrade arterial access, a diagnostic angiogram was performed. A 0.014 glidewire advantage guidewire and a 0.035 Navicross catheter (Terumo inc) were again used to cross the proximal calcified stenotic posterior tibial artery. Afterward, a 3.5 mm S4 IVL catheter (Shockwave medical) was used for the treatment of the proximal part of the posterior tibial not only to improve the inflow to the pDVA but also to change the compliance of the vessel wall at the crossing point. After successful IVL treatment, the pDVA was performed as previously described (Figure 2). The patient was discharged at the third post-procedural day, and no major events were observed during the first 30 days. A secondary procedure (scoring balloon and DCB angioplasty) was performed because of a stenosis of the vein foot arch 3 months after the initial procedure which was detected during follow-up in the duplex ultrasound examination. A second angioplasty of the vein arch was indicated 5 months after the index revascularization due to recurrence of the stenosis (Figure 3) also detected in the regular duplex ultrasound. No inflow stenosis was observed. At 2 years follow-up, the reconstruction remains patent and the patient is asymptomatic.

Both patients provided written informed consent prior to the procedure, and this paper is in accordance with the Declaration of Helsinki.

## Discussion

pDVA provides a revascularization alternative in no-option CLTI patients. The procedure aims to establish a retrograde venous flow, bypassing the arterial occlusion and providing arterial pedal perfusion.^8^ IVL is an effective and safe modality for the treatment of heavily calcified femoropopliteal and tibial disease; however, its use prior to pDVA has never been described.^9,10^ This report presents the combination of both treatment strategies within limb salvage procedures in patients with heavily calcified, non-reconstructable distal PAD. IVL was used to optimize the inflow to the arteriovenous circuit and enable the crossing from the tibial arteries to the vein, while pDVA provided arterial flow to an otherwise occluded arterial foot arch.

Several studies assessed the performance of pDVA. The ALPS registry evaluated 32 patients who underwent pDVA with a reported 1-year amputation-free survival (AFS) of 71% and a 68.2% rate of complete wound healing.^11^ In the PROMISE I Study, 32 patients were evaluated with a 70% AFS and 75% of wound healing at 1 year. Nonetheless, re-intervention rates were high in both studies (54.8% and 52%, respectively).^12^ More recently, the PROMISE II trial enrolled 105 patients who had non-reconstructable CLTI. A limb salvage was attained in 67 patients, and wounds were completely healed in 16 of 63 patients (25%) and were in the process of healing in 32 of 63 patients (51%). Again, re-interventions to treat both native vessels and to optimize the flow were performed in 38 patients (36.5%).^7^

Zaman et al proposed five different patterns of DVA failure (failure types) based on the location of the lesion. Most of the patients presented with multiple types of failure concurrently.^13^ The use of IVL for calcified lesions of the arterial inflow and especially of the popliteal artery and the TPT may improve the patency of the circuit and eventually avoid an inflow re-intervention.^13^ Additionally, IVL was used to change the compliance of the vessel wall and enable an easier crossing, which can be technically demanding.

Although several options can be used for the treatment of calcified disease, the use of IVL has several advantages. It provides a “leave nothing behind” option in a vascular territory prone for restenosis, while the low rates of periprocedural perforation and distal embolization secure the preservation of the collateral network.

## Conclusion

The selected use of IVL during pDVA could improve the patency of this reconstruction in no-option patients, while reducing the risk of inflow re-intervention.
